# Experience with a third-generation parathyroid hormone assay (BIO-PTH) in the diagnosis of primary hyperparathyroidism in a Brazilian population

**DOI:** 10.1590/2359-3997000000183

**Published:** 2016-08-23

**Authors:** Teresa Cristina P. Bonanséa, Monique Nakayama Ohe, Cynthia Brandão, Cláudia de Francischi Ferrer, Lívia Marcela Santos, Marise Lazaretti-Castro, José Gilberto Henriques Vieira

**Affiliations:** 1 Escola Paulista de Medicina Universidade Federal de São Paulo São Paulo, SP Brasil Divisão de Endocrinologia, Escola Paulista de Medicina, Universidade Federal de São Paulo (EPM-Unifesp), São Paulo, SP, Brasil; 2 Laboratório Fleury São Paulo SP Brasil Laboratório Fleury, São Paulo, SP, Brasil

**Keywords:** Primary hyperparathyroidism, Bio-PTH, intact PTH, PTH assays

## Abstract

**Objective:**

To evaluate the usefulness of a third-generation PTH assay in the diagnosis of primary hyperparathyroidism (PHPT).

**Subjects and methods:**

Forty-one PHPT patients (4 men and 37 women) with 61.2 ± 10.9 (mean ± SD) years, were studied and had PTH levels measured with two different methods using the same immunochemiluminescent assay plataform (Elecsys 2010 System, Roche). We compared a second-generation assay (I-PTH) with a third-generation PTH assay (Bio-PTH). Two populations of 423 and 120 healthy adults with serum 25OHD levels above 25 ng/mL were used to define normal values in the I-PTH and Bio-PTH assays respectively.

**Results:**

Normal PTH values based in the healthy adults population were 24.2-78.0 pg/mL for the I-PTH assay and 19.9-58.5 pg/mL for Bio-PTH assay. In PHPT patients, PTH values ranged from 67 to 553 pg/mL (median: 168 pg/mL) using the I-PTH assay and from 55 to 328 pg/mL (median: 111 pg/mL) using the Bio-PTH assay. Results obtained with the Bio-PTH assay were significantly lower (p < 0.0001, Wilcoxon). In general I-PTH and Bio-PTH showed highly significant correlation (r = 0.952, p < 0.0001). Passing–Bablok analysis gave a regression equation of Bio PTH = 13.44 + 0.59 x intact PTH. PHPT patients had 25OHD levels ranging from 4 to 36 ng/mL (mean 16.2 ng/mL); 35 subjects (85.3%) had values bellow 25 ng/mL.

**Conclusion:**

Our results demonstrate that both second and third generation PTH methods are strongly correlated in PHPT patients and control subjects. Lower results with Bio-PTH tests are expected in function of the assay specificity determined by the amino-terminal antibody used.

## INTRODUCTION

Parathyroid hormone (PTH) is an 84-amino-acid peptide produced exclusively by the parathyroid glands with a major role in regulating serum levels of calcium (1). Biochemically, this peptide contains two main portions, one carboxyl-terminal (COOH) and one amino-terminal (NH_2_). The classic biological activity of PTH, mediated by its receptor (type 1 PTH receptor, PTHR1), is dependent on the presence of an intact amino-terminal sequence, including the first four amino acids (2,3). After PTH is synthesized, it is stored in vesicles and partially metabolized within the cells. In this process, amino-terminal fragments are degraded, and carboxyl-terminal fragments are secreted along with intact molecules (PTH 1–84). These latter forms are quickly metabolized in the liver and have a half-life of fewer than 4 minutes. The carboxyl-terminal fragments are eliminated by glomerular filtration and have a longer half-life in the circulation, although their biological effects are not completely known (4,5). These fragments have an activity potentially independent from their binding to the PTHR1 receptor which, according to some studies, can result in hypocalcemia, hypophosphatemia, and increase in urinary phosphorus (6). Thus, circulating forms of PTH are quite heterogeneous and, considering that immunoassays depend on sequences recognized by antibodies, measurement of the so-called intact molecule (PTH 1-84) becomes a challenge (4).

The first radioimmunoassay (RIA) for human PTH was described by Berson and cols. in 1963 and, since then, several different assays have emerged (3,7). The first RIA used polyclonal antibodies generated against PTH from bovine or porcine parathyroid glands, with specificity mainly directed against the carboxyl-terminal region (4,8). They were also called first-generation assays, and their major limitation was interference from PTH carboxyl fragments, especially in patients with impaired renal function (8). These characteristics imposed on these assays low sensitivity and specificity rates, in addition to significantly different results obtained with these first-generation methods (3).

Immunometric assays were introduced in the late 1980s. These second-generation assays were based on the binding of two distinct antibodies, one against the amino-terminal segment and another against the carboxyl-terminal portion of the PTH, simplifying the diagnostic process (9). However, the ability of second-generation assays to measure the intact PTH 1–84 form alone has been questioned after human studies showed the existence of long carboxyl-terminal fragments with a partial deletion of the first amino acids in the amino-terminal portion, such as the form 7–84 (10-12). Thus, levels of biologically active PTH could be overestimated when measured by second-generation assays, since these long fragments are recognized by these assays even though they do not activate the PTHR1 (12).

Third-generation immunometric assays (Bio-PTH) have been developed in an attempt to identify exclusively the biologically active PTH (1–84) form. An amino-terminal specific antibody directed to the first four amino acids is used in third-generation assays to restrict the measurement to active molecular forms (1–84), not recognizing, therefore, long carboxyl-terminal fragments (3,9).

Primary hyperparathyroidism is the third most common endocrine disorder, with an asymptomatic presentation in most cases (13). Measurement of serum PTH levels is crucial in establishing the diagnosis of primary hyperparathyroidism, although the measurement may be normal or inconclusive in some patients (14). To make matters worse, primary hyperparathyroidism may also present as a normocalcemic disease (15). Considering these factors, we conducted this study to evaluate the contribution of a third-generation assay in improving the diagnosis of primary hyperparathyroidism.

## SUBJECTS AND METHODS

We studied 41 patients with primary hyperparathyroidism, followed up at the Metabolic Bone Diseases Unit of a teaching hospital at *Escola Paulista de Medicina* (Unifesp). The diagnosis of primary hyperparathyroidism had been confirmed by an elevated concentration of serum calcium measured at least twice, and intact PTH levels above the upper normal limit or inappropriately high using a second-generation assay (I-PTH, Elecsys 2010 System, Roche), which is the standard method used at our center. Blood samples were collected after an overnight fast and aliquots were instantly frozen at -70^o^C. Serum calcium, phosphorus, albumin, and creatinine were measured by standard chemistry. Serum 25-hydroxyvitamin D [25(OH)D] was measured by chemiluminescence using the Liaison^®^ (CLIA) assay.

All subjects had biochemical data consistent with the disease and were symptomatic or meeting at least one criteria for parathyroidectomy according to guidelines for the management of asymptomatic primary hyperparathyroidism from the Fourth International Workshop (16). Among these subjects, 29 underwent subsequent parathyroidectomy until the end of the study. Pathological analyses of their surgical specimens revealed that 25 of these patients had adenomas and four had parathyroid hyperplasia. Creatinine clearance, estimated with the Cockcroft-Gault equation (17), was above 60 mL/min in all patients. We measured PTH levels in the entire cohort with a second-generation (I-PTH) and a third-generation assay (Bio-PTH) using the same blood sample. Both measurements were performed in the same immunochemiluminescent assay platform (Elecsys 2010 System, Roche). We used in both assays a capture antibody specific to the carboxyl-terminal section, around amino acids 37–42. Two distinct amino-terminal antibodies were used for each assay, each one binding at two different antigenic sites: the 26–32 portion in the I-PTH assay and the 1–6 region in the Bio-PTH assay.

Two groups of normal adults living in the city of São Paulo, one with 423 individuals and another with 120, were used as controls to estimate normal PTH values measured with the I-PTH and Bio-PTH assays, respectively. All subjects in these groups had 25(OH)D levels above 25 ng/mL and normal calcium levels.

### Statistical analysis

For statistical analyses, we used the software GraphPad Prism, version 5 (GraphPad Software, Inc., La Jolla, CA, USA) or MedCalc, version 12 (Medcalc Software bvba, Mariakerke, Belgium). Data are expressed as mean and standard deviation (SD), and 95% confidence interval (CI) for normally-distributed data, or as median and range for non-normally distributed data. The agreement between assays was assessed using Passing-Bablok regression and Bland-Altman analysis. Correlation was performed using Pearson’s correlation coefficient. The results were considered significant when their *p* values were < 0.05.

## RESULTS

The mean age of the 41 patients with primary hyperparathyroidism enrolled in the study was 61.2 ± 10.9 years, and 37 were women. Serum PTH levels in the control group varied from 24.2–78.0 pg/mL when measured with the I-PTH assay and 19.9–58.5 pg/mL when measured with the Bio-PTH assay. The biological data of the patients with primary hyperparathyroidism are shown in Table 1.

**Table 1:** Biological data of 41 patients with primary hyperparathyroidism

Variable	Mean ± SD	Normal range
Age (years)	61.2 ± 10.9	N/A
25-OHD (ng/mL)	16.2 ± 7.2	30-60 ng/mL
Serum calcium (mg/dL)	11.6 ± 0.9	8.6-10.2
Ionized calcium (mmol/L)	1.61 ± 0.15	1.24-1.41
Serum phosphorus (mg/dL)	2.6 ± 0.4	2.5-4.5
Serum creatinine (mg/dL)	0.9 ± 0.3	0.5-1.2

SD: standard deviation; N/A: not available; 25(OH)D: 25-hydroxyvitamin D.

In patients with primary hyperparathyroidism, the mean serum concentration of calcium was 11.6 ± 0.9 mg/dL (normal range 8.6–10.2 mg/dL) and that of ionized calcium was 1.61 ± 0.15 mmol/L (normal range 1.24–1.41 mmol/L). PTH values ranged from 67 to 553 pg/mL (median 168 pg/mL) when measured with the intact assay (I-PTH) and from 55 to 328 pg/mL (median 111 pg/mL) when measured with the Bio-PTH assay. Levels of 25(OH)D in patients with primary hyperparathyroidism ranged from 4 to 36 ng/mL (mean 16.2 ng/mL) and in 35 of the 41 subjects (85.3%), the levels were below 25 ng/mL.

Among patients with primary hyperparathyroidism, serum PTH levels were elevated in 36 (87.8%) when measured with the I-PTH assay and in 38 (92.7%) when measured with the Bio-PTH assay. All subjects with normal PTH levels by both assays had other causes of hypercalcemia excluded.

**Figure 1:**
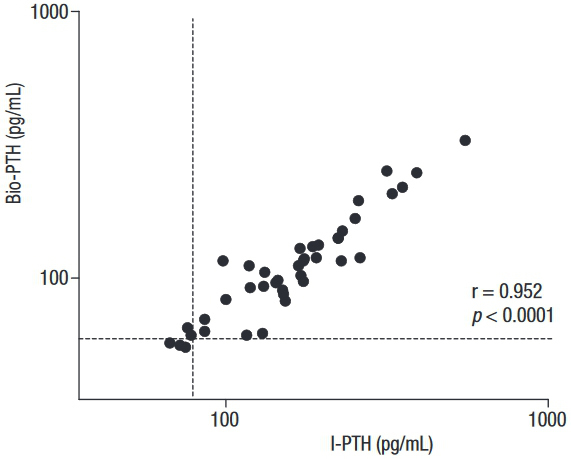
Correlation between serum Bio-PTH versus I-PTH in patients with primary hyperparathyroidism.

**Figure 2:**
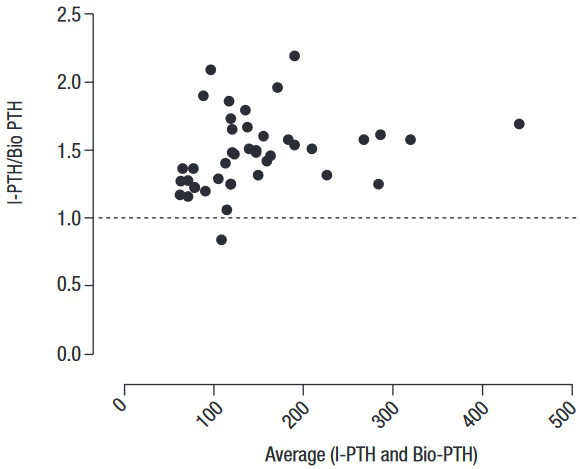
Bland-Altman plot of the ratio of PTH levels obtained with the I-PTH and Bio-PTH assays (I-PTH/Bio-PTH ratio) against the average PTH level obtained with both methods. The bias ± 2 standard deviations (SD) between the methods was 1.5 ± 0.27.

Regression analysis showed that I-PTH and Bio-PTH were highly correlated (r = 0.952, *p* < 0.0001) (Figure 1). However, the Passing-Bablok analysis resulted in the regression equation Bio-PTH = 13.44 + 0.59 x intact PTH. The 95%CI of the slope did not include 1 (95% CI 0.55–0.66), and that of the intercept did not include 0 (95% CI 2.08–20.0), indicating the presence of a proportional and systematic error. A Bland-Altman plotting also demonstrated poor agreement between the methods, with an increasing difference within the higher range of measurements (Figure 2).

Final numeric values obtained with the Bio-PTH assay were significantly lower than those obtained with the I-PTH assay (*p* < 0.0001, Wilcoxon), although one patient had a lower PTH value when measured by I-PTH compared with the value measured with Bio-PTH.

## DISCUSSION

Primary hyperparathyroidism is a common endocrine disease. Over the past decades, the disease has shown a change in presentation at diagnosis, from very symptomatic to oligo- or asymptomatic, even in developing countries (13,18). These patients, who present with mild primary hyperparathyroidism, are less likely to show abnormal laboratory findings at diagnosis, and present serum calcium and PTH measurements not as elevated as patients with overt symptoms of the disease (19).

The introduction of second-generation assays has improved the quality of PTH measurement in all conditions associated with changes in this hormone’s levels. However, with an accumulation of data in the past years, the use of second-generation assays has shown some limitations (2). Since this assay recognizes PTH using two different antibodies, it is supposed to measure only whole PTH (1–84), the form that is capable of binding and activating the PTHR1 (3,9,12). Amino-terminal antibodies generally used in this assay target the most antigenic portion of the amino nucleus, which lies between amino acids 15–26 (9). However, Amour’s and cols. were the first to demonstrate the existence of long PTH fragments, such as PTH (7–84), that may be identified by second-generation assays since only the first amino acid in the molecule is missing (10). The occurrence of these long PTH fragments is probably frequent, mainly in patients with kidney diseases due to their decreased glomerular filtration rate. Their occurrence has also been described in healthy individuals and patients with primary hyperparathyroidism (1,9). Previous studies have suggested increased PTH degradation within the glands in the presence of hypercalcemia and, consequently, a higher proportion of PTH fragments in the circulation (8,9). However, this phenomenon is not clinically important in patients with primary hyperparathyroidism. Brossard and cols. have compared the response to calcium infusion in healthy individuals and patients with hyperparathyroidism and found no significant difference in levels of carboxyl-terminal fragments between these two groups (20).

Due to the strict identification of PTH 1–84 and no measurement of its fragments, the absolute value of PTH measured with a third-generation assay is expected to be lower than that with a second-generation assay (9). This correlates with our findings in the current study. The use of a specific antibody targeting the first amino acid of the molecule prevents fragmented forms from interfering in the measurement (21). The ratio of third- to second-generation assays for PTH is usually < 1 in primary hyperparathyroidism, but in rare cases, the serum concentration of PTH measured with a third-generation assay may be higher than that measured with a second-generation assay, yielding a > 1 ratio (21,22). A possible explanation is that these patients may produce forms of PTH molecules that are not detected by second-generation assays and are possibly recognized by third-generation ones. Amour and cols. recently described an amino PTH (N-PTH), a molecule with preservation of the four first amino acids, therefore, recognizable by third-generation assays. However, N-PTH presents changes in the portion between amino acids 15–20 that prevents its recognition by second-generation assays (10,14). The main hypothesis is that this N-PTH molecule has 84 amino acids, but its serine residue 17 may be phosphorylated, which modifies the binding site of the amino-terminal antibody used in the I-PTH assay (10). This situation is more likely to be found in severe hyperparathyroidism and parathyroid carcinomas, although it has already been described in seven patients with primary hyperparathyroidism without any clinical or histological signs of parathyroid carcinoma (14). In our cohort with primary hyperparathyroidism, one patient showed a higher PTH value using the Bio-PTH assay compared with the I-PTH.

In our cohort with primary hyperparathyroidism, the I-PTH and Bio-PTH assays identified elevated PTH levels in 87.8% and 92.7% of the patients, respectively. Aligned with findings by other authors, we found a high correlation between the two methods in these patients (23-25). However, the Passing-Bablok and Bland-Altman tests showed a proportional and systematic difference between both methods, indicating that the second and third-generation PTH assays may not be used interchangeably.

In the literature, there are only a few studies evaluating third-generation PTH assays in patients with primary hyperparathyroidism (8,23-25). Silverberg and cols. have found significantly higher sensitivity with third-generation assays in a population with primary hyperparathyroidism (8). The explanation for this superior diagnostic specificity may be related to the use of different control groups to define the normal PTH values for each assay, especially regarding potential differences in vitamin D status in these populations (8,14,25). Populations with different vitamin D levels may have been used to establish the normal range for each assay (14). Also, many other factors may interfere with PTH values in addition to vitamin D status, including calcium and magnesium intake, gender, age, and ethnicity (14,26). Although we used two different populations in our study to estimate the normal range for each assay, all controls had 25(OH)D levels greater than 25 ng/mL. This may explain the absence of differences in diagnostic sensitivity between both assays, which is consistent with findings also reported by several other authors (23-25)*.*

One remarkable finding in our patients with primary hyperparathyroidism was their low levels of 25(OH)D (mean 16 ng/mL). Patients with primary hyperparathyroidism have been described as having vitamin D deficiency when compared with individuals in the normal population (27). The mechanisms behind this finding are not well understood, but one of the factors involved may be a greater PTH conversion of 25(OH)D to 1,25-dihydroxyvitamin D [1,25(OH)_2_D]. Furthermore, the subsequent increase in 1,25(OH)_2_D levels stimulate the inactivation of 25(OH)D by 24-hydroxylases (27).

In conclusion, serum PTH results correlated well when measured with a third-generation assay (Bio-PTH) and a second-generation assay (I-PTH). We found that the use of a third-generation assay was not superior to that of a second-generation assay in the diagnosis of primary hyperparathyroidism. Nevertheless, the assays are different and should not be used interchangeably. Although we evaluated patients with primary hyperparathyroidism with an indication for surgery and high levels of serum calcium, this assay could be potentially helpful also in suspicious cases of primary hyperparathyroidism with borderline PTH values and mild disease. Another aspect to be explored in relation to the assays is their intraoperative use. Since the half-life of PTH 1–84 is slightly shorter than that of PTH fragments, the assays may help identify an early decrease in PTH levels (4).
